# 

**Published:** 1910-10-22

**Authors:** 


Telegraphic Address: Ospedale, London.

THE MODERN MEDICAL AND INSTITUTIONAL JOURNAL.

Telephone Number: 2734 Gerrard.

NEW SERIES. No. 193, Vol. VIII. No. 1265, Vol. XLIX. | SATURDAY, OCT. 22ND, 1910. | PRICE THREEPENCE

